# Enhancing treatment of osteoarthritis knee pain by boosting expectancy: A functional neuroimaging study

**DOI:** 10.1016/j.nicl.2018.01.021

**Published:** 2018-02-28

**Authors:** Jian Kong, Zengjian Wang, Jaclyn Leiser, Domenic Minicucci, Robert Edwards, Irving Kirsch, Ajay D. Wasan, Courtney Lang, Jessica Gerber, Siyi Yu, Vitaly Napadow, Ted J. Kaptchuk, Randy L. Gollub

**Affiliations:** a Department of Psychiatry, Massachusetts General Hospital, Charlestown, MA, USA; b MGH/MIT/HMS Athinoula A. Martinos Center for Biomedical Imaging, Charlestown, MA, USA; c Department of Anesthesiology, Perioperative, and Pain Medicine, Brigham and Women's Hospital and Harvard Medical School, Boston, MA, USA; d Department of Psychiatry, Brigham and Women's Hospital and Harvard Medical School, Boston, MA, USA; e Program in Placebo Studies, Beth Israel Deaconess Medical Center, Harvard Medical School, Boston, MA, USA; f Department of Anesthesiology, University of Pittsburgh School of Medicine, Pittsburgh, PA, USA; g Department of Psychiatry, University of Pittsburgh School of Medicine, Pittsburgh, PA, USA

**Keywords:** Knee osteoarthritis, Expectancy, Acupuncture, Reward, Resting state functional connectivity

## Abstract

**Objectives:**

Expectation can significantly modulate pain and treatment effects. This study aims to investigate if boosting patients' expectancy can enhance the treatment of knee osteoarthritis (KOA), and its underlying brain mechanism.

**Methods:**

Seventy-four KOA patients were recruited and randomized to three groups: boosted acupuncture (with a manipulation to enhance expectation), standard acupuncture, or treatment as usual (TAU). Each patient underwent six treatments before being debriefed, and four additional treatments after being debriefed. The fMRI scans were applied during the first and sixth treatment sessions.

**Results:**

We found significantly decreased knee pain in the boosted acupuncture group compared to the standard acupuncture or TAU groups after both six and ten treatments. Resting state functional connectivity (rsFC) analyses using the nucleus accumbens (NAc) as the seed showed rsFC increases between the NAc and the medial prefrontal cortex (MPFC)/rostral anterior cingulate cortex (rACC) and dorsolateral prefrontal cortex in the boosted group as compared to the standard acupuncture group after multiple treatments. Expectancy scores after the first treatment were significantly associated with increased NAc-rACC/MPFC rsFC and decreased knee pain following treatment.

**Conclusions:**

Our study provides a novel method and mechanism for boosting the treatment of pain in patients with KOA. Our findings may shed light on enhancing outcomes of pharmacological and integrative medicines in clinical settings.

## Highlights

• Acupuncture with enhanced expectancy produced greater pain relief in KOA patients.
• NAc – ACC/MPFC rsFC increased after acupuncture with enhanced expectancy.
• NAc – ACC/MPFC rsFC increases are associated with clinical improvements.
• Our findings provide a novel method for boosting the treatment of chronic pain.

## 1 Introduction

Non-specific effects, such as the placebo effect, play an important role in medical practice (Finniss et al., 2010; Price et al., 2008). Under certain circumstances, such as clinical trials, it presents challenges for investigators. In other circumstances, it can enhance treatment outcomes (Gollub and Kong, 2011; Weiss and Swede, 2016). While the placebo effect is well accepted, there is still much to learn about its underlying mechanism and how to harness it in clinical settings.

It is believed that expectation plays an important role in non-specific effects, particularly in the placebo effect (Amanzio and Benedetti, 1999; Amanzio et al., 2013; Atlas et al., 2012; Tracey, 2010). Investigators have found a well-accepted expectancy manipulation model (Eippert et al., 2009; Hashmi et al., 2014; Kong et al., 2006, Kong et al., 2009a; Wager et al., 2004), in which they surreptitiously reduce stimulus intensity after placebo treatment to make subjects believe the treatment is effective, that can produce greater placebo effects compared to verbal suggestion alone (Colloca et al., 2008; Kong et al., 2013b; Voudouris et al., 1990). In addition, this model can also enhance the effect of active treatments in healthy volunteers (Bingel et al., 2011; Kong et al., 2009a). Nevertheless, few studies have applied the expectancy manipulation model on the chronic pain patient population due to the difficulty in manipulating chronic pain intensity compared to experimental pain. In this study, we first applied an expectancy model using experimental heat pain to enhance subjects' expectation of acupuncture analgesia, and then tested whether this enhanced expectation improved the treatment effect of acupuncture on chronic pain caused by knee osteoarthritis (OA).

Although still under investigation, one potential neural mechanism by which enhanced expectation may lead to improved therapeutic outcomes is through the engagement of the reward system in the brain. Using pain as an example, the expectation of treatment effect (pain relief) can be rewarding and pleasurable, thus expectation, in the context of treatment in clinics, can be regarded as a special case of reward (Leknes and Tracey, 2008; Petrovic et al., 2005; Scott et al., 2007; Yu et al., 2014b). In support of this hypothesis, neuroimaging studies have found that the reward system, particularly the nucleus accumbens (NAc), is involved in mediating placebo effects in patients with Parkinson's disease (de la Fuente-fernandex et al., 2002), depression (Mayberg et al., 2002), anxiety (Petrovic et al., 2005), and pain (Scott et al., 2007; Yu et al., 2014b).

Literature suggests that two neurotransmitter systems are involved in the pain modulation of reward expectation and motivation: the dopamine system increases motivation, whereas the opioid system influences motivation indirectly by modulating subjective feelings of pain and reward (Berridge, 2007; Navratilova et al., 2015a; Navratilova and Porreca, 2014). Studies also found that the two systems are closely related neuroanatomically, and interact in complex ways (Leknes and Tracey, 2008). The brain regions that are particularly well-situated to mediate interactions between the two systems are the NAc (Schmidt et al., 2014; Smith and Berridge, 2007; Szechtman et al., 1981; Wei et al., 2004; Zubieta et al., 2001), anterior cingulate cortex (ACC), and medial prefrontal cortex (MPFC) (Hare et al., 2008; Navratilova et al., 2015a, Navratilova et al., 2015b; Tuominen et al., 2012).

Recent studies have demonstrated that resting state functional connectivity (rsFC) can provide information about the intrinsic functional organization of the brain (Fox and Raichle, 2007; Vincent et al., 2006), improve our understanding of pain modulation (Kong et al., 2013a), and predict treatment outcomes (Tetreault et al., 2016). In this study, we investigated 1) if boosted expectancy of acupuncture analgesia for experimental pain can enhance acupuncture treatment for knee OA and 2) how boosted expectancy modulates rsFC of the NAc. We chose acupuncture treatment of chronic pain because it provides an excellent model for studying the modulation effect of expectation. Studies indicate that the non-specific effect of acupuncture is robust (Cherkin et al., 2009; Vickers et al., 2012). Furthermore, acupuncture is gaining popularity due to its total clinical effectiveness (Berman et al., 2010; Vickers and Linde, 2014), and rarity of adverse effects (Melchart et al., 2004; White et al., 2001). We hypothesized that acupuncture with boosted expectancy would 1) produce greater clinical improvements than acupuncture alone and 2) increase rsFC between the NAc and rACC/MPFC.

## 2 Materials and methods

### 2.1 Subjects

Subjects with knee OA were recruited. Experiments were conducted with approval from the Massachusetts General Hospital Institutional Review Board and with the written, informed consent of each participant. All subjects agreed to allow their data to be analyzed. The study was registered at clincaltrials.gov (NCT#: 01040754).

Inclusion criteria included: between 40 and 70 years of age; met the Classification Criteria of the American College of Rheumatology for osteoarthritis of the right and/or left knee; radiographic evidence of Grade 2 or 3 knee OA using the Kellgren-Lawrence Scale. Exclusion criteria were: interventional procedure for knee pain within two months, including corticosteroid injections to the knee; intent to undergo surgery during the time of involvement in the study; presence of a cardiovascular, neurological or psychiatric disorder; additional pain disorder with severity greater than knee OA pain; pregnancy; acupuncture treatment within one year; difficulties reading, speaking or understanding English. All subjects were told to maintain their baseline medications and other treatments for their knee OA during the duration of the study. They were prompted to report any changes in treatment, including frequency of prn medications, at each study visit.

### 2.2 Experimental procedure

Subjects were stratified by gender and the most affected knee, and then randomized into one of three groups: boosted acupuncture, standard acupuncture, or treatment as usual (TAU control) at the beginning of session 2 (Supplementary Fig. 1). The randomization table was created by a study biostatistician using the R program. Both acupuncture groups received identical acupuncture treatments for four weeks (2 times/week for the first two weeks, and 1 time/week for the last two weeks). After a mid-point evaluation and debriefing, patients received an additional 4 weeks of acupuncture (1 time/week).

All subjects in the two acupuncture groups participated in a total of 13 study visits, including the baseline training and clinical assessment (session 1), first fMRI scan session including the first acupuncture treatment (session 2), four acupuncture treatments (session 3–6), second fMRI scan session including the sixth acupuncture treatment (the procedure was identical to the first MRI scan) and debriefing at the end of the session in the boosted acupuncture group (session 7), midpoint clinical assessment (session 8), 4 additional acupuncture treatments over the course of a month (session 12), and final clinical assessment (session 13) (Supplementary Fig. 1). In the boosted acupuncture group, an expectancy manipulation similar to our previous studies (Hashmi et al., 2014; Kong et al., 2006, Kong et al., 2009a, Kong et al., 2009b) was applied during the fMRI scan sessions (during treatment one and treatment six) to enhance subjects' positive expectation of pain reduction with acupuncture treatment.

The TAU group participated in 5 visits, including the baseline training and assessment, first fMRI scan, second fMRI scan, midpoint assessment, and a final assessment. They followed the exact timing for the acquisition of behavioral, clinical and imaging data, but without any treatment (Supplementary Fig. 1).

Session 1 was a training and baseline clinical outcome measurement session. Following being screened and signing the consent form, all subjects completed the Knee Injury and Osteoarthritis Outcomes Score (KOOS) to measure their knee pain and function.

A 1 × 3 grid was drawn on the medial surface of the affected knee, avoiding the patella. Then, calibrated thermal heat pain stimuli were delivered to the medial side of the affected knee using a PATHWAY system with a 3 cm × 3 cm probe (Egorova et al., 2015b; Egorova et al., 2015c). Each stimulus was initiated at a 32 °C baseline and increased to a target temperature that was presented for 12 s, including 2.5 s to ramp up and ramp down. The inter-stimulus interval ranged from 24 to 30 s. Subjects rated their pain for each stimulus during the inter-stimulus interval using a 0–20 Gracely Sensory Box scale (Gracely et al., 1978a, Gracely et al., 1978b).

Similar to our previous studies (Hashmi et al., 2014; Kong et al., 2006, Kong et al., 2009a, Kong et al., 2009b), subjects first experienced one ascending series of calibrated heat stimuli. The first stimulus of each ascending series was initiated from a target temperature of 38°C. Subsequent stimuli were increased by 1 °C to 52 °C or to the subject's tolerance: a rating of ~17 (Very Intense) on the Gracely Scale. Two temperatures, one that elicited low ratings (5–7; mild to moderate) and one that elicited high ratings (14–15; strong) were selected for each subject. Once the two temperatures for a subject were determined, he or she was tested for rating response consistency. Random sequences of 4 low and 4 high intensity noxious stimuli were administered. The temperatures were further adjusted as needed. Subjects had to consistently rate the high intensity stimuli as greater than the low intensity stimuli to continue in the study.

Session 2 was an fMRI scan session that included a resting state fMRI scan applied at the beginning of the session, followed by an expectancy manipulation in the boosted group and first acupuncture treatment for those in the acupuncture groups.

### 2.3 Expectancy manipulation

Subjects' expectation that acupuncture treatment would relieve their experimental pain was manipulated in the boosted group using similar methods as employed in our previous studies in healthy subjects and subjects with chronic pain due to knee OA (Hashmi et al., 2014; Kong et al., 2006, Kong et al., 2009a, Kong et al., 2009b). At the beginning of the session, subjects were given a scripted explanation that a person's responses to acupuncture can be positive or neutral, and that this response tends to remain consistent over time. They were also told that previous studies had shown that if acupuncture relieved pain induced by experimental heat that would indicate they were more likely to have a good outcome for their endogenous knee pain over the course of the treatments.

**Fig. 1:**
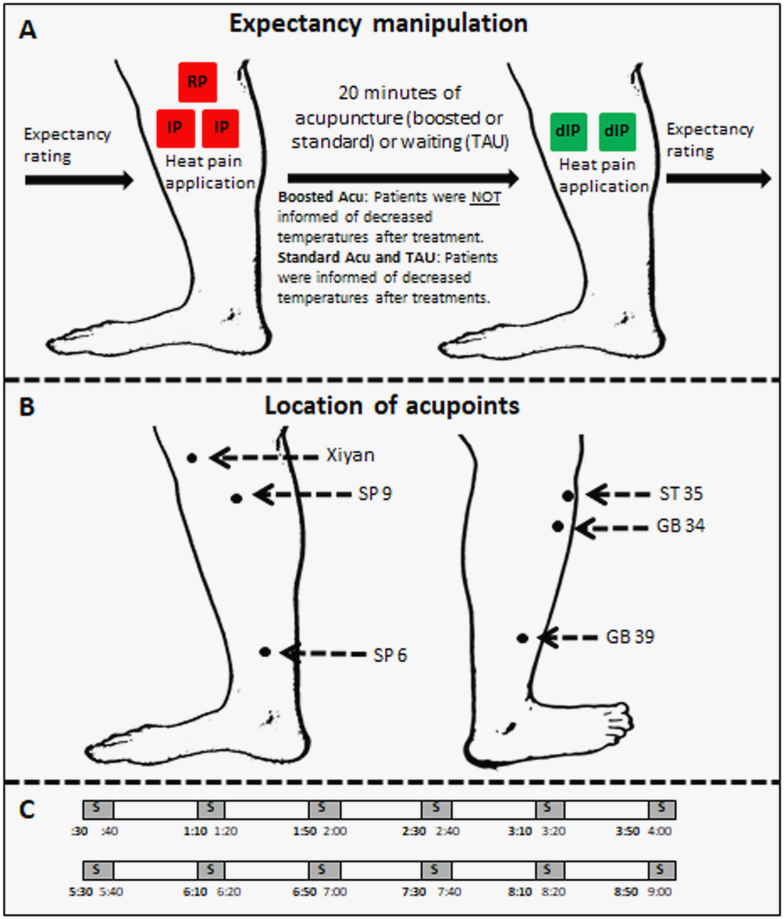
A: Details of expectancy manipulation paradigm. We first used a marker to draw three boxes identical in size on the medial side of the most affected knee. Then, we placed the thermal probe in one of the boxes at a time. One box received random pain (RP), consisting of four moderate intensity pain stimuli and four low intensity pain stimuli in a random order. RP stimuli were applied to ensure subjects could consistently rate pain stimuli. The other two boxes received identical pain (IP) sequences consisting of 6 identical moderate pain stimuli. After acupuncture treatment (boosted or standard) or waiting for 20 min (TAU group), decreased identical pain sequences (dIP), consisting of 6 identical low intensity pain stimuli, were applied on all the two IP boxes in the same order as above. Those in the boosted acupuncture group were informed they were going to receive identical pain stimuli similar to before the treatment to test the analgesic effect of acupuncture. Those in the other groups (standard and TAU) were informed they were going to receive lowered intensity stimuli to investigate brain responses to different levels of pain. After this manipulation, patients in the boosted acupuncture group felt acupuncture significantly relieved the heat pain. Expectancy scores were collected before and after expectancy manipulation. B: Locations of 6 acupoints. C: Stimulation parameters applied for acupuncture treatment. This procedure was applied twice. S, needle stimulation.

Neither of these statements is in fact known to be true. This verbal suggestion is one component of the manipulation to enhance expectation for analgesia. The conditioning aspect of the expectancy manipulation consisted of telling subjects that they would receive identical heat pain stimuli before and after treatment, but in reality, after treatment we surreptitiously lowered heat pain temperatures in the boosted group to reduce their rating to “faint to weak” and hence give subjects an unmistakable experience of profound analgesia (Fig. 1A).

Subjects in the standard acupuncture group experienced the same pain as the boosted group, but were told the truth at the beginning of the session and reminded before the post-treatment pain application right after acupuncture treatment that we would administer pain stimuli of a lower intensity so that we could investigate their brain response to a different level of pain (Fig. 1A). As a result, they should feel less pain, but this has no relation to the treatment effect of acupuncture. The same information was given to the TAU control group. Identical procedures were repeated in the sixth treatment, during the second MRI scan for each group (Fig. 1A).

All subjects rated their expectation of how acupuncture treatment would modify the intensity of the pain they experienced in response to calibrated, experimental heat pain stimuli and endogenous knee pain at baseline (session 1), before and after expectancy manipulations during the first and second scans, and at the end of the study (session 13) (Fig. 1A). They used the same 0–10 visual analogue scale (0 indicates “does not work at all”, and 10 indicates “complete pain relief”), labeled explicitly to assess expected relief of exogenous heat pain or endogenous chronic knee pain.

To explore whether the effects of this manipulation to boost expectation of pain relief would persist if subjects were aware of what we did, we debriefed the boosted subjects at the end of the second scan (sixth treatment, session 7). To debrief the subjects, we read them a script explaining that we reduced the heat pain stimuli intensity after their treatment to give them an unmistakable experience of analgesia. We also assured them that if they experienced clinical improvements in knee pain, this pain relief was real and might well continue.

### 2.4 Acupuncture administration

All subjects in the two acupuncture groups received 10 identical acupuncture treatments over two months according to the following schedule: 2 times/week for the first two weeks and 1 time/week for the last six weeks.

The 6 acupoints selected for treatment were ST35, Xi yian (extra point), GB34, SP9, GB39 and SP6 (Fig. 1B). This set of acupuncture points are most commonly used in clinical treatment trials for knee pain (Berman et al., 2004; Scharf et al., 2006; Witt et al., 2005). In subjects with bilateral knee pain, the treatments were applied to the knee with the most pain or the knee the subject believed needed more attention.

We followed identical procedures as in our previous experiment (Chen et al., 2015; Chen et al., 2014; Egorova et al., 2015a; Spaeth et al., 2013). During acupuncture treatment, the acupuncturist stimulated one point at a time in a predetermined order, each for 10 s with 30-second breaks between each acupoint (Fig. 1C). Each acupoint was stimulated 4 times in a treatment. We randomized the specific starting acupoint across subjects, but held it constant throughout all sessions for each individual subject. For consistency, we kept leg position, acupoint location, and needling parameters (1–2 cm depth, approximately 120 rotations per minute, and moderate *deqi* sensations on a 0–10 scale) constant across the two groups. In the fMRI sessions, there were two 9-min acupuncture scans per treatment followed by an assessment with the Massachusetts General Hospital Acupuncture Sensation Scale (MASS) (Kong et al., 2007a; Spaeth et al., 2013).

### 2.5 Clinical outcomes and data analysis

#### 2.5.1 Knee injury and Osteoarthritis Outcome Score (KOOS)

We measured clinical outcomes using the KOOS (Roos and Toksvig-Larsen, 2003) measured at baseline, the midpoint, and the end of the study. KOOS is comprised of 5 subscales: 1) pain, 2) other symptoms, 3) function in daily living (ADL), 4) function in sports and recreation, and 5) knee-related quality of life (QOL). Each subscale allows for the calculation of a normalized score, with 0 denoting the most extreme symptoms/pain and 100 denoting no symptoms/pain (Roos and Toksvig-Larsen, 2003). Based on previous studies (Berman et al., 2004; Chen et al., 2015; Egorova et al., 2015a; Spaeth et al., 2013), we selected the KOOS pain subscale as our primary outcome measure, and other subscales as our secondary outcome measures. Trained research assistants administered the KOOS to all subjects.

Clinical outcome analysis was performed using SPSS 18.0 Software (SPSS Inc., Chicago, IL, USA). The ANCOVA analysis was applied to compare the change in KOOS pain subscales and other scores across the three groups respectively. Age and gender were included in the model. ANOVA analysis was applied to compare the expectancy scores measured at different points. A post hoc analysis (Sidak) was applied to explore post-hoc between-group differences. Multiple regression analyses were applied to explore the association between the expectancy score, resting state functional connectivity changes, and KOOS pain subscore changes following treatments across all patients who received acupuncture treatments.

### 2.6 fMRI data acquisition and data analysis

Whole brain imaging was performed with a 3-Tesla Siemens MRI system. For the BOLD scans during resting state (6 min), the acquisition included 47 slices with a thickness of 3 mm, a TR of 3000 ms, a TE of 30 ms, flip angle of 85 degrees, field of view of 216 mm^2^ and a 3 × 3-mm in-plane spatial resolution. Subjects were instructed to keep their eyes opened during the resting fMRI scan. Structural images were also obtained by a MP-RAGE sequence (TR = 2530 ms, TE = 1.69, echo time = 9.8 ms, flip angle of 7, field-of-view of 256 mm, slice thickness = 1 mm). Due to the characteristics of knee OA, patients usually do not experience knee pain while they are lying still in the scanner.

Similar to our previous studies (Liu et al., 2016; Wang et al., 2016 #4392; Song et al., 2017; Tao et al., 2017; Tao et al., 2016; Wang et al., 2017), functional connectivity analysis was carried out by applying a seed-based approach using the CONN toolbox (Whitfield-Gabrieli and Nieto-Castanon, 2012) [http://www.nitrc.org/projects/conn]. Bilateral NAc templates extracted from the IBASPM (atlas71) using WFU-Pick Atlas software (Maldjian et al., 2003) were selected as the seed (region of interest).

The preprocessing of fMRI data was performed using Statistical Parametric Mapping (SPM8) (Wellcome Department of Cognitive Neurology, University College, London, UK) in MATLAB (Mathworks, Inc., Natick, Massachusetts) incorporated into the CONN toolbox. The preprocessing steps included slice-timing correction, realignment, co-registration to subjects' respective structural images, which was used to normalize images to the standard Montreal Neurological Institute template, and smoothing with an 8 mm full width at half maximum (FWHM) kernel. In addition to these steps, we employed segmentation of gray matter, white matter, and cerebrospinal fluid (CSF) areas for the removal of temporal confounding factors (white matter and CSF) (Whitfield-Gabrieli and Nieto-Castanon, 2012). Band-pass filtering was performed with a frequency window of 0.008–0.09 Hz.

To eliminate head motion and artifacts, we identified outlier time points in the motion parameters and global signal intensity using ART (http://www.nitrc.org/projects/artifact_detect). For each subject, we treated images (time points) as outliers if composite movement from a preceding image exceeded 0.5 mm, or if the global mean intensity was >3 standard deviations from the mean image intensity for the entire resting scan. Outliers were included as regressors in the first level general linear model along with motion parameters. Four subjects were excluded due to over-determined models (no degrees of freedom for this subject) as suggested by ART.

First-level correlation maps were produced by extracting the residual BOLD time course from each NAc seed and by computing Pearson's correlation coefficients between that time course and the time courses of all other voxels in the brain. Correlation coefficients were Fisher transformed into ‘Z’ scores to increase normality and allow for improved second-level General Linear Model analyses.

Whole brain second level group analysis was applied using two sample *t*-tests to compare the NAc functional connectivity changes between different groups. Similar to our previous studies (Wang et al., 2017; Liu et al., 2016; Wang et al., 2016; Song, 2017; Tao et al., 2017; Tao et al., 2016), a threshold of voxel-wise *p* < 0.005 uncorrected and cluster-level *p* < 0.05 family-wise error (FWE) correction was applied for all fMRI data analysis.

## 3 Results

### 3.1 Clinical outcomes

Of the 74 subjects who participated in the study, twenty subjects were excluded (Supplementary Fig. 1). Five subjects chose to leave the study because they decided their schedules were incompatible. One subject was excluded due to an atypical pain response; he was insensitive to even the highest temperature setting on the MEDOC. Six subjects left the study after discovering they were claustrophobic at the time of the scan. Two subjects decided they did not want their knees exposed to any heat following their screening visit. Three subjects decided not to participate after learning they would be in the waitlist group. Finally, three subjects were lost to follow up before their first MRI scans. Twelve of the twenty subjects who dropped had been randomized, with five assigned to the boosted acupuncture group, five assigned to the TAU group, and two assigned to the standard acupuncture.

There was no systematic reason that influenced subjects to withdraw from the study and there was no significant difference in dropout rates among the three groups (*p* = 0.29). 54 subjects aged 60.2 +/- 7.5 years (19 boosted acupuncture, 18 standard acupuncture, and 17 TAU controls) completed both fMRI scans. Four subjects (1 standard acupuncture and 3 TAU controls) were excluded from the rsFC analysis due to excessive motion. Four additional subjects' KOOS data was missing (2 boosted and 2 TAU controls) from the measurements taken at midpoint. The final analysis was applied only to subjects with complete data and thus 46 subjects were included in the fMRI and KOOS pain score data analysis.

**Fig. 2:**
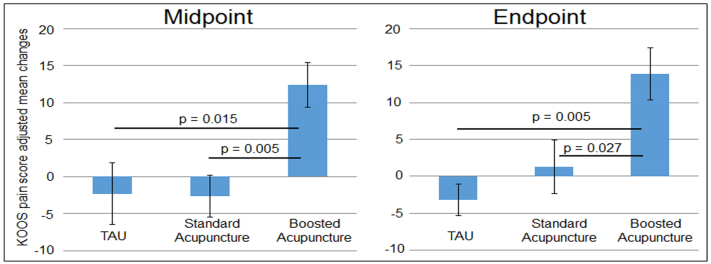
KOOS pain score changes measured at the midpoint and the end of the study compared to baseline (mean ± SE).

The ANCOVA analysis of change in the KOOS pain subscale scores at baseline and midpoint showed significant differences among the three groups when gender and age were controlled for, F_(2,41)_ = 6.97, *p* = 0.002 (Fig. 2). Post hoc analysis indicated that the boosted acupuncture significantly decreased pain compared to standard acupuncture (*p* = 0.005) and TAU control groups (*p* = 0.015). In addition, we also found significant differences between the boosted acupuncture and standard acupuncture groups in KOOS Sport subscale scores, and between boosted acupuncture and TAU groups. There was no significant difference between the standard acupuncture and TAU control (Table 1).

**Table 1:** Demographics and KOOS scores (mean + SD). Higher KOOS scores indicate less pain. ADL, activities of daily living. QOL, Quality of Life. TAU, treatment as usual.

Characteristic	Boosted Acu	Standard Acu	TAU
Age	61.3 ± 6.9	61.2 ± 7.7	60.1 ± 7.1
N (Female/Male)	17 (9/8)	17 (10/7)	12 (8/4)
Baseline			
KOOS Pain	57.7 ± 13.9	68.6 ± 13.2	63.7 ± 15.6
KOOS Symptoms	57.6 ± 15.2	64.1 ± 12.4	68.7 ± 16.9
KOOS ADL	64.4 ± 18.2	75.7 ± 17.6	76.1 ± 16.3
KOOS Sport	35.3 ± 25.3	52.1 ± 25.1	43.3 ± 26.5
KOOS QOL	32.7 ± 18.9	43.4 ± 19.2	46.9 ± 17.4
Midpoint after 6 treatments			
KOOS Pain	70.1 ± 15.6	66.0 ± 13.4	61.3 ± 15.2
KOOS Symptoms	64.7 ± 14.8	62.2 ± 14.5	65.2 ± 19.2
KOOS ADL	73.5 ± 15.0	75.7 ± 12.8	71.2 ± 15.7
KOOS Sport	52.6 ± 25.9	50.9 ± 19.0	49.2 ± 23.1
KOOS QOL	47.1 ± 16.8	51.8 ± 10.5	50.0 ± 17.5
Endpoint after 10 treatments			
KOOS Pain	71.4 ± 14.7	69.9 ± 15.5	60.4 ± 14.7
KOOS Symptoms	66.0 ± 17.2	65.5 ± 19.4	64.3 ± 19.3
KOOS ADL	74.7 ± 15.1	79.6 ± 16.0	69.7 ± 16.5
KOOS Sport	44.7 ± 25.4	59.1 ± 25.6	46.2 ± 28.7
KOOS QOL	47.4 ± 19.1	54.0 ± 17.8	47.4 ± 16.7
Change between baseline and midpoint			
KOOS Pain	12.4 ± 12.4	−2.6 ± 11.6⁎	−2.3 ± 14.4⁎
KOOS Symptoms	7.1 ± 14.2	−1.9 ± 12.8	−3.6 ± 11.9
KOOS ADL	9.2 ± 13.5	0.0 ± 11.7	−4.9 ± 12.1⁎
KOOS Sport	17.4 ± 23.7	−1.2 ± 19.7⁎	5.8 ± 18.8
KOOS QOL	14.3 ± 14.6	8.5 ± 15.3	3.1 ± 17.8
Change between baseline and endpoint			
KOOS Pain	13.9 ± 14.4	1.3 ± 14.9⁎	−3.2 ± 7.4⁎
KOOS Symptoms	8.4 ± 16.8	1.5 ± 16.7	−4.5 ± 11.3
KOOS ADL	10.3 ± 15.9	3.9 ± 15.2	−6.4 ± 6.4⁎
KOOS Sport	9.4 ± 34.4	7.1 ± 19.5	2.9 ± 13.9
KOOS QOL	14.7 ± 13.6	10.7 ± 17.6	0.5 ± 15.6⁎

⁎ Indicates significant difference in change when compared to boosted acupuncture group.

The ANCOVA analysis of change in KOOS pain subscale scores at baseline and the end of the study (after 10 treatments) also showed significant differences among the three groups, F_(2,41)_ = 5.56, p = 0.005 (Fig. 2). Post hoc analysis indicated that the boosted treatment significantly decreased pain compared to standard acupuncture (*p* = 0.027) and TAU control groups (p = 0.005). In addition, we also found significant differences between the boosted acupuncture and TAU group in KOOS ADL and QOL subscale scores. There were no significant differences between standard acupuncture and TAU control groups (Table 1).

Subjects' expectancy of acupuncture analgesia in response to experimental heat pain and endogenous knee pain were measured at baseline, before and after expectancy manipulation during the first and second scans, and at the end of the study (Table 2). We found significant group differences in expectancy scores following the expectancy manipulation during the first scan for both heat pain (F_(2,42)_ = 7.04, p = 0.002; post hoc analysis: Boosted vs Standard Acupuncture *p* = 0.041, Boosted vs TAU *p* = 0.003; Standard vs TAU *p* = 0.477) and knee pain (F_(2,42)_ = 4.46, *p* = 0.018; post hoc analysis: Boosted vs Standard Acupuncture *p* = 0.025, Boosted vs TAU *p* = 0.091; Standard vs TAU *p* = 0.99). There was no significant difference between groups in expectancy scores at other time points (Table 2).

**Table 2:** Expectancy (mean (SD)) at different time points across three treatment groups (*n* = 46).

Modalities	Groups	Baseline Visit	First Scan		Second Scan		Last Visit
			Pre	Post	Pre	Post	
Heat pain expectancy	Boosted Acu	6.1 (1.8)	5. 8 (2.8)	7.5 (1.8)	6.1 (2.8)	7.2 (2.4)	5.2 (3.2)
	Standard Acu	5.6 (2.1)	5.2 (2.9)	5.4 (2.7)⁎	5.2 (2.9)	5.4 (3.2)	4.5 (2.6)
	TAU	6.1 (2.6)	4.5 (3.3)	4.1 (2.9)⁎	5.5 (2.6)	5.1 (2.3)	5.3 (2.9)
Knee pain expectancy	Boosted Acu	6.9 (1.8)	6.7 (2.1)	7.9 (1.8)	6.9 (2.3)	7.5 (2.2)	6.4 (2.7)
	Standard Acu	6.2 (2.1)	5.3 (2.8)	5.7 (2.5)⁎	5.4 (2.8)	5.9 (2.7)	5.6 (2.6)
	TAU	6.1 (1.4)	6.1 (2.8)	5.9 (2.7)⁎	5.9 (2.3)	5.4 (2.1)	5.8 (2.4)

⁎ Indicates a significant difference in change when compared to boosted acupuncture group.

**Fig. 3:**
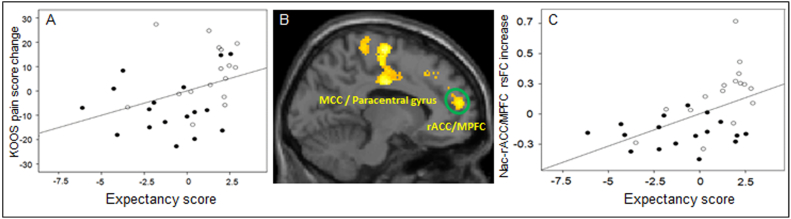
A. Partial plot showing the positive association between the expectancy scores after the expectancy manipulation in session 2 (x axis) and KOOS pain increases (clinical improvement) after six acupuncture treatments (y axis), including age and gender as covariates (*p* = 0.04) across the two acupuncture groups (solid dot indicates standard acupuncture group, hollow dot indicates boosted acupuncture group). B. Representative brain regions showed a significant rsFC increase in boosted acupuncture as compared to standard acupuncture (voxel-wise *p* < 0.005, and cluster-level *p* < 0.05 FWE corrected). C. The scatter plot represents the partial plot showing the positive association between the expectancy score (x axis) after expectancy manipulation in session 2 and NAc and rACC/MPFC connectivity changes (y axis), including age and gender as covariates (*p* = 0.002). MCC, middle cingulate cortex; rACC, rostral anterior cingulate cortex; MPFC, medial prefrontal cortex.

To explore the general association between the expectancy at the beginning of treatment can influence clinical outcomes after multiple treatments, we also performed a regression analysis between the expectancy scores for how treatment impacts endogenous knee pain measured after the first expectancy manipulation, and clinical improvement (change in KOOS pain score) after six and ten treatments. Since the two acupuncture groups received identical acupuncture treatment, to increase the power, we pooled the data from the two groups. We found a significant association between expectancy scores and clinical improvements (*p* = 0.04) (Fig. 3A) after six treatments, but not after ten treatments (*p* = 0.15).

Based on the medication logs, only one subject (from the standard acupuncture group) reported a medication change. That subject developed a toothache and took ibuprofen as recommended by her dentist. The subject's KOOS pain score was 72.2 before the first scan and 72.2 after the sixth treatment, thus she showed no improvement as measured by the KOOS pain scale.

### 3.2 Functional connectivity results

A comparison of NAc rsFC changes (‘post’ minus ‘pre’) between the boosted and standard acupuncture treatments revealed a significant increase of rsFC between the NAc and left middle cingulate cortex (MCC)/paracentral gyrus (PaCG)/postcentral gyrus (PoCG), left medial prefrontal cortex (MPFC)/rostral anterior cingulate cortex (rACC), right PoCG and left dorsolateral prefrontal cortex (DLPFC) in the boosted acupuncture group (Table 3, Fig. 3B). Boosted acupuncture treatment produced greater rsFC increases between the NAc and left ventral medial prefrontal cortex (vMPFC) compared to the TAU group (Table 3). There were no NAc rsFC differences in any brain region between the standard acupuncture and TAU groups at the threshold we set.

**Table 3:** NAc rsFC change (post-treatment minus pre-treatment) differences among the three treatment groups.

Comparison	Brain Region	Cluster Size	MNI Peak (X, Y, Z)			Z Value
Boosted > Standard	Left MCC	1027	−12	−12	36	4.54
	Left paracentral gyrus		−10	−16	66	4.09
	Left postcentral gyrus		−34	−42	72	3.89
	Left MPFC/rACC	695	−4	40	32	4.48
	Right postcentral gyrus	445	4	−42	80	3.69
	Left DLPFC	749	−30	18	26	3.68
Standard > Boosted	None					
Boosted > TAU	Left MPFC	616	−6	40	32	4.88
TAU > Boosted	None					
Standard > TAU	None					
TAU > Standard	None					

Given our hypothesis of the important role of NAc and MPFC/rACC in the rewarding effect of pain relief (Navratilova et al., 2015a), we also extracted the cluster value of NAc – MPFC/rACC rsFC increases (scan 2 – scan 1), and explored if the expectancy score at the beginning of the treatment can modulate the NAc – MPFC/rACC resting state functional connectivity changes after multiple acupuncture treatments by calculating the association between the two. Additionally, we also investigated the association between the NAc – MPFC/rACC resting state functional connectivity changes and the corresponding KOOS pain changes after repeated treatments, including age and gender as covariates. Since our aim is to explore the general relationship, rather than a specific treatment group, between resting state functional connectivity, expectancy scores at the beginning of the treatment, and clinical outcome changes, it is appropriate to pool the data from the two groups that received identical treatment in order to increase power. We found a significant association between 1) the NAc – MPFC/rACC rsFC increases and the expectancy scores (*p* = 0.002) (Fig. 3C) and 2) the NAc – MPFC/rACC rsFC increases and KOOS pain rating increases (clinical improvement) after six treatments (*p* = 0.04).

## 4 Discussion

In this study, we explored the feasibility of enhancing acupuncture treatment on knee OA using a well-tested expectancy manipulation model. We found that boosted acupuncture with enhanced expectation of pain relief can significantly increase acupuncture's therapeutic effect on knee pain compared to standard acupuncture which received identical acupuncture treatment as boosted acupuncture group. We also found that boosted expectation significantly enhanced the rsFC of the NAc with rACC, MPFC, DLPFC, and paracentral and postcentral gyri. Expectancy scores at the beginning of the study were significantly associated with NAc-rACC/MPFC rsFC increases and with KOOS pain score increases (clinical improvement) after repeated acupuncture treatments across all subjects who received acupuncture treatments.

We found for the first time that boosted expectation of pain relief to experimental heat pain due to an active treatment (acupuncture) can be transferred to chronic knee pain to enhance clinical outcomes. Knee OA is a major age-related public health problem and a leading cause of long-term pain and disability (Murphy et al., 2008; Murray et al., 2012). Pharmacological treatment of knee OA is often ineffective with unwanted and dangerous side effects (Berman et al., 2004; Felson et al., 2000). Arguably, acupuncture may be a promising treatment option for knee OA due to its effectiveness in relieving pain (Berman et al., 2004; Hinman et al., 2014a; Mavrommatis et al., 2012; Scharf et al., 2006; Suarez-Almazor et al., 2010; Witt et al., 2005), and the rarity of adverse effects (MacPherson et al., 2001; Melchart et al., 2004; Ochi, 2013; White et al., 2001). Our findings will shed new light on how to enhance the acupuncture treatment of this highly prevalent disorder and other chronic pain disorders.

We found enhanced clinical improvement even after being debriefed, which persisted at least for one month after being debriefed. This finding is consistent with a previous study investigating the post-debriefing effect using experimental pain (Wickless and Kirsch, 1989). It is also consistent with a more recent study suggesting that placebo analgesia to experimental pain may be produced by prior conditioning rather than by current expected pain relief (Schafer et al., 2015). Our results suggest that once a treatment is thought to be effective, whether there was manipulation to enhance expectation or not, experiencing the improvement may produce a conditioning-like effect.

When we combined all subjects who received acupuncture, subjects' expectation of acupuncture to treat their knee pain after the first treatment was significantly associated with clinical improvement after six acupuncture treatments, implying the important role of expectation in acupuncture treatment. This result is consistent with clinical studies suggesting that baseline expectancy is associated with pain improvement after acupuncture treatment (Kalauokalani et al., 2001; Linde et al., 2007). For instance, in a pooled analysis of four trials of acupuncture in patients with migraine, tension-type headache, chronic low back pain, and knee OA (Linde et al., 2007), Linde and colleagues found a significant association between clinical improvement and greater expectations for a good outcome. In another study investigating the effects of acupuncture and communication style in patients with knee OA (Suarez-Almazor et al., 2010), investigators found that there was no significant difference between the real and sham acupuncture groups, while high expectation communication styles (using positive utterances such as “I think this will work for you”) did have a small but significant effect on pain reduction and satisfaction in knee pain control as compared to a neutral expectation communication style (treatment conveyed with statements of uncertainty such as “this may or may not work for you”). Nevertheless, some clinical trials failed to find the association between expectancy ratings and therapeutic effects of acupuncture on chronic pain (Sherman et al., 2010; Thomas et al., 2006), suggesting that the situation is complex. Further studies with larger sample sizes are needed to further replicate our findings.

We only observed slightly greater improvements (not significant) in standard acupuncture as compared to TAU controls after 10 acupuncture treatments. This may be due to the small sample size in this study. The result is consistent with a recent acupuncture clinical trial on knee OA in which the authors found that acupuncture treatments only produced mild improvements in knee pain compared with no acupuncture controls (Hinman et al., 2014b). This result is also in line with a recent osteoarthritis care and management guideline that does not recommend acupuncture treatment for OA (https://www.nice.org.uk/guidance/cg177/chapter/1-Recommendations#non-pharmacological-management-2). Most importantly, our results endorse the robustness of expectation in modulating treatment effects, and its potential to improve clinical practice.

Multiple neurocognitive constructs have been used to theorize about potential mechanisms for how expectation can modulate one's pain experience. One such construct works through the reward circuitry, which includes the dopaminergic pathways from the NAc and its connections with the opioid rich ACC (Navratilova et al., 2015a). Pain and reward are both powerful motivators of behavior that share neuroanatomical substrates (Leknes and Tracey, 2008). Pain is usually considered to be the opposite of pleasure; thus, relief of pain can be regarded as a special case of reward. Scott and colleagues (Scott et al., 2007) found that the release of dopamine from the NAc plays a crucial role in the expectancy modulation of pain. Wanigasekera et al. (Wanigasekera et al., 2012) found that individuals with high reward responsiveness, a personality trait dependent on endogenous opioid neurotransmission, experience more exogenous remifentanil-induced behavioral analgesia. Becker and colleagues found that pain relief engages endogenous pain-inhibitory pathways when obtained in a motivated state, which might be used to improve pain treatment by a positive and self-amplifying feedback loop (Becker et al., 2015).

The role of the reward/motivation circuitry in mediating enhanced treatment outcomes due to expectation of analgesia is also supported by a study in which (Lee et al., 2015) acupuncture needle stimulation was associated with the verbal suggestion that treatment will be effective. This association with positive verbal suggestion produced greater fMRI signal increases at the ventral striatum, including the NAc, as compared to pure needle stimulation given without the verbal suggestion. In another study, Li and colleagues (Li et al., 2016) found rsFC changes between the PAG and the NAc, as well as the MPFC/rACC before and after one-month of acupuncture treatment were significantly associated with migraine headache intensity relief. Although we did not observe the NAc and PAG connectivity changes at the threshold we set, we found increased NAc – PAG and surrounding area increases in the boosted acupuncture group compared to standard acupuncture group at a liberal threshold (*p* < 0.05 voxel-wise). We speculate this is due to the relatively small sample size of this study. Taken together, these studies support the important role of the NAc in expectancy modulation and acupuncture treatment.

In our study, we found significant rsFC increases between the NAc and MPFC/rACC. Studies suggest that the rACC/MPFC is a key region in the descending pain modulatory system (Eippert and Tracey, 2014; Fields, 2004; Jensen et al., 2012; Kong et al., 2013a; Kucyi et al., 2013; Tracey et al., 2002; Yu et al., 2014a), forming a core network with the PAG and rostral ventral medulla (RVM) (Kong et al., 2010). Studies show that the rACC/MPFC is involved in the self-regulation of pain such as placebo analgesia (Petrovic et al., 2002 #1445; Kong et al., 2006 #1775; Eippert et al., 2009 #2373; Kong et al., 2007a, Kong et al., 2007b #2006; Kong et al., 2013a, Kong et al., 2013b #2921) and acupuncture treatment of chronic pain (Chen et al., 2015; Chen et al., 2014; Egorova et al., 2015a; Li et al., 2016) and depression (Wang et al., 2016). A recent study (Baliki et al., 2012) found that greater functional connectivity of the NAc with the MPFC/ACC can predict the development of sub–acute back pain patients. A more recent study (Brown et al., 2015) found that compared to healthy controls, patients with arthritis pain had overall less opioid receptor availability within the striatum. Previous studies propose that the MPFC/rACC, supported by the putamen, provides an expected value-related input to the PAG, which then conveys aversive prediction error signals to regulate behavior (Eippert and Tracey, 2014; Roy et al., 2014). Thus, enhanced NAc and rACC/MPFC rsFC suggests that boosted expectancy may work by strengthening the descending pain modulation system.

There are several limitations in this manuscript. First, we did not measure the knee pain intensity during the fMRI scan systemically. Nevertheless, all patients reported they did not experience or only experienced very mild knee pain while they were lying in the scanner. Secondly, we did not include the duration of the patients' knee pain in the manuscript since most patients could not give an accurate starting time of the knee pain. We suspect our randomization adjusted for this potential confound.

In conclusion, we found that the boosted expectation of acupuncture analgesia in response to experimental pain can be transferred to clinical pain, which can in turn enhance the acupuncture treatment effect for chronic pain in patients with knee OA. Boosted expectation significantly strengthens the rsFC between the reward/motivation system, descending pain modulation system, and sensory and affective pain processes to enhance the acupuncture treatment effect. Elucidation of these brain processes may shed light on how to enhance treatment outcomes of pharmacological and various integrative medicines in clinical settings.

The following is the supplementary data related to this article.

Supplementary Fig. 1 Flow chart of the study. Supplementary Fig. 1
