# Supplementary figures and images for: Enhancing treatment of osteoarthritis knee pain by boosting expectancy: A functional neuroimaging study
Source: Neuroimage Clin. 2018 Feb 28;18:325–34. doi: 10.1016/j.nicl.2018.01.021 (PMC5984593; doi:10.1016/j.nicl.2018.01.021)

Supplementary Figure 1. Flow chart of the study

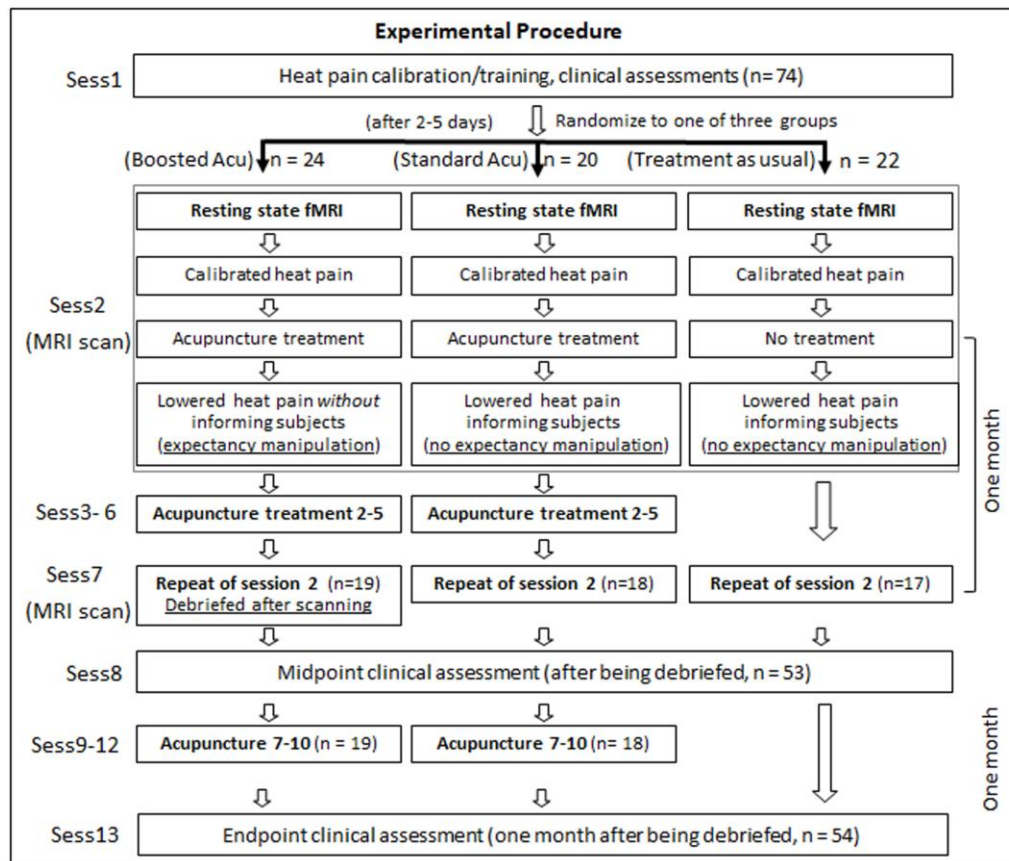

Supplement: Supplementary Fig. 1 — Flow chart of the study. [file mmc1.pdf]
